# Cost-Effectiveness of Population-Based Multigene Testing for Breast and Ovarian Cancer Prevention

**DOI:** 10.1001/jamanetworkopen.2023.56078

**Published:** 2024-02-14

**Authors:** Fangjian Guo, Victor Adekanmbi, Christine D. Hsu, Abbey B. Berenson, Yong-Fang Kuo, Ya-Chen Tina Shih

**Affiliations:** 1 Department of Obstetrics and Gynecology, The University of Texas Medical Branch at Galveston, Galveston; 2 Center for Interdisciplinary Research in Women’s Health, The University of Texas Medical Branch at Galveston, Galveston; 3 Department of Biostatistics and Data Science, The University of Texas Medical Branch at Galveston, Galveston; 4 Office of Biostatistics, University of Texas Medical Branch at Galveston, Galveston; 5 Program in Cancer Health Economics Research, Jonsson Comprehensive Cancer Center, and Department of Radiation Oncology, School of Medicine, University of California, Los Angeles

## Abstract

**Importance:**

The current method of *BRCA* testing for breast and ovarian cancer prevention, which is based on family history, often fails to identify many carriers of pathogenic variants. Population-based genetic testing offers a transformative approach in cancer prevention by allowing for proactive identification of any high-risk individuals and enabling early interventions.

**Objective:**

To assess the lifetime incremental effectiveness, costs, and cost-effectiveness of population-based multigene testing vs family history–based testing.

**Design, Setting, and Participants:**

This economic evaluation used a microsimulation model to assess the cost-effectiveness of multigene testing (*BRCA1, BRCA2, *and* PALB2*) for all women aged 30 to 35 years compared with the current standard of care that is family history based. Carriers of pathogenic variants were offered interventions, such as magnetic resonance imaging with or without mammography, chemoprevention, or risk-reducing mastectomy and salpingo-oophorectomy, to reduce cancer risk. A total of 2000 simulations were run on 1 000 000 women, using a lifetime time horizon and payer perspective, and costs were adjusted to 2022 US dollars. This study was conducted from September 1, 2020, to December 15, 2023.

**Main Outcomes and Measures:**

The main outcome measure was the incremental cost-effectiveness ratio (ICER), quantified as cost per quality-adjusted life-year (QALY) gained. Secondary outcomes included incremental cost, additional breast and ovarian cancer cases prevented, and excess deaths due to coronary heart disease (CHD).

**Results:**

The study assessed 1 000 000 simulated women aged 30 to 35 years in the US. In the base case, population-based multigene testing was more cost-effective compared with family history–based testing, with an ICER of $55 548 per QALY (95% CI, $47 288-$65 850 per QALY). Population-based multigene testing would be able to prevent an additional 1338 cases of breast cancer and 663 cases of ovarian cancer, but it would also result in 69 cases of excess CHD and 10 excess CHD deaths per million women. The probabilistic sensitivity analyses show that the probability that population-based multigene testing is cost-effective was 100%. When the cost of the multigene test exceeded $825, population-based testing was no longer cost-effective (ICER, $100 005 per QALY; 95% CI, $87 601-$11 6323).

**Conclusions and Relevance:**

In this economic analysis of population-based multigene testing, population-based testing was a more cost-effective strategy for the prevention of breast cancer and ovarian cancer when compared with the current family history–based testing strategy at the $100 000 per QALY willingness-to-pay threshold. These findings support the need for more comprehensive genetic testing strategies to identify pathogenic variant carriers and enable informed decision-making for personalized risk management.

## Key Points

**Question:**

Is a population-wide genetic testing strategy more cost-effective than the current family history–based testing strategy for breast and ovarian cancer prevention?

**Findings:**

This economic evaluation found that population-based *BRCA1, BRCA2, *and* PALB2* testing among unselected women was cost-effective for the prevention of breast and ovarian cancer and remained cost-effective in extensive 1-way sensitivity analyses. Population-wide genetic testing was 100% cost-effective for all the simulations in probabilistic sensitivity analyses; it became cost-inefficient only when the cost of the test exceeded a certain threshold ($825).

**Meaning:**

The findings support the need for a shift toward more comprehensive genetic testing strategies to identify pathogenic variant carriers and enable informed decision-making for personalized risk management.

## Teaser

This economic evaluation examines the cost-effectiveness of population-based multigene testing compared with family history–based testing in simulated patients with breast and ovarian cancer.

## Introduction

The discovery in the 1990s of *BRCA1 *(OMIM 113705) and *BRCA2* (OMIM 600185) mutations in women susceptible to breast or ovarian cancer made targeted, individualized cancer preventive care possible.^1^ Germline *BRCA* mutations account for approximately 5% to 10% of breast cancer and 10% to 18% of ovarian cancer.^2,3,4,5^ Women who carry pathogenic variants of *BRCA* genes may undergo magnetic resonance imaging (MRI) with or without mammography screenings for breast cancer, which can lead to early diagnosis and timely treatment. They may also consider risk-reducing options, such as chemoprevention with selective estrogen receptor modulators or aromatase inhibitors, or risk-reducing mastectomy (RRM) to further decrease their risk of developing breast cancer. Additionally, they may opt for risk-reducing salpingo-oophorectomy (RRSO) as a preventive measure to reduce their risk of ovarian cancer. Mutations in many other DNA mismatch repair genes besides *BRCA1/2* have also been linked to high breast cancer or ovarian cancer susceptibility.^6,7^ Next-generation sequencing allows for testing of tens of genes simultaneously. Multigene tests^8^ bundle testing for *BRCA* mutations with other high-penetrance genes, providing more information on clinically actionable genes.

The current method of testing for *BRCA* gene mutations,^6,9,10,11,12,13^ which is based on family history, often fails to identify many carriers. Family history–based testing will miss more than 50% of pathogenic variant carriers,^14,15,16^ which leaves a large number of women who are at risk for hereditary breast or ovarian cancer undiagnosed and untreated.^17^ In fact, approximately 70% of *BRCA* pathogenic variant carriers with breast or ovarian cancer and 95% of unaffected carriers remain unidentified in the US.^18^ This finding underscores the need for more comprehensive genetic testing strategies to identify pathogenic variant carriers. Although population-based multigene testing is an attractive alternative preventive strategy to family history–based testing strategy, this unselected testing strategy can be costly. In this study, we used a state-transition microsimulation model to evaluate the cost-effectiveness of population-based multigene testing compared with family history–based testing as a means of preventing hereditary breast and ovarian cancer.

## Methods

A health economic analysis plan was developed for this economic evaluation.^19^ This study, conducted between September 1, 2020, and December 15, 2023, was exempted from full board review by the institutional review board at The University of Texas Medical Branch at Galveston, and informed consent was not required for the use of deidentified data. This report follows the Consolidated Health Economic Evaluation Reporting Standards (CHEERS) 2022 reporting guideline for economic evaluations, decision analytic models, or simulated modeling studies.^20^

We developed a microsimulation model to assess the cost-effectiveness of multigene testing for all women aged 30 to 35 years compared with the current standard of care that is family history based (TreeAge Pro Healthcare, version 2023). We selected *BRCA1, BRCA2,* and *PALB2* (OMIM 610355) genes for analyses because they are the most prevalent high-penetrance genes^21,22^ and are all associated with high breast cancer susceptibility.^23^ Screening with MRI and RRM is now offered to pathogenic variant carriers of these genes. Our model did not include other high-risk genes (*CDH1, PTEN,* and *TP53*) because they are very rare, and pathogenic variants in these genes confer only a relatively low relative risk of developing breast cancer. Many health care professionals believe that these genes do not meet the clinical intervention threshold, and National Comprehensive Cancer Network guidelines do not recommend early initiation of breast cancer screening or RRM for carriers of pathogenic variants in these genes.^24^

**Figure 1.**
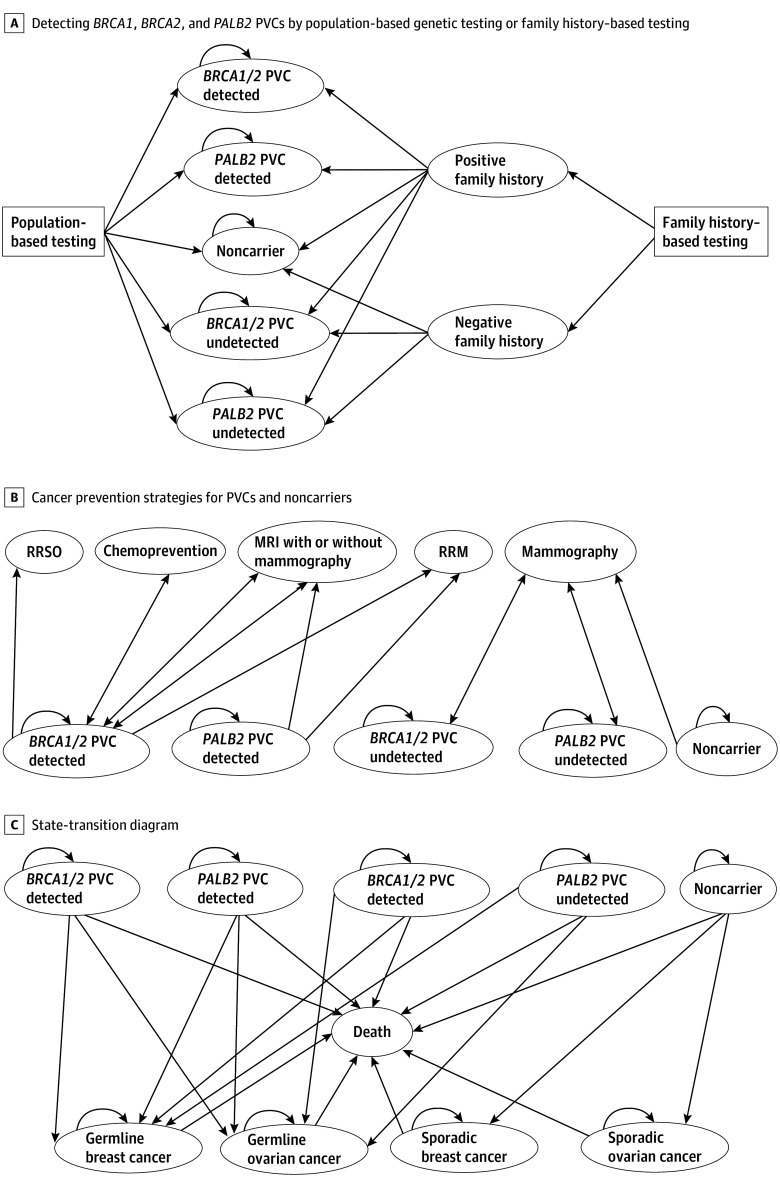
Schematic Diagram of the Microsimulation Model Structure We assumed a genetic test uptake rate of 0.7. MRI with or without mammography indicates enhanced screening for breast cancer with MRI with or without mammography. Mammography indicates general screening for breast cancer with mammography. MRI indicates magnetic resonance imaging; PVC, pathogenic variant carrier; RRM, risk-reducing mastectomy; RRSO, risk-reducing salpingo-oophorectomy.

We show a schema of the microsimulation model about unaffected women for the development of breast and ovarian cancer in Figure 1 and the eFigure in Supplement 1. The microsimulation model enables the incorporation of diverse genetic types and age variations at the individual level while allowing for tracing an individual’s disease history in cases where prior events impact subsequent cycles. We assume all eligible women in the population-based testing arm and those meeting family history–based testing criteria in the family history–based testing arm receive genetic counseling. We assumed a test uptake rate of 70% for both arms and performed a 1-way sensitivity analysis on this modeling parameter. All tested individuals are offered posttest genetic counseling, including noncarriers. Without counseling, noncarriers may decrease mammography screening adherence, leading to missed early detection opportunities. Only approximately 10% of breast cancer cases are hereditary, so noncarriers still face a risk of developing cancer. Counseling after negative results can provide guidance on appropriate screening.

Identified carriers of pathogenic variants are offered enhanced screening with MRI with or without mammography. Additionally, identified carriers with *BRCA1*/*BRCA2* pathogenic variants are offered options of chemoprevention, RRM, and RRSO, and those identified with *PALB2* pathogenic variants are offered RRM. We assumed that the median ages at which women carrying a pathogenic variant would opt for RRM and RRSO are 37 and 40 years, respectively. We also conducted a scenario analysis with older median age at RRM (42 years) and RRSO (46 years) based on previous reports.^25,26^ We accounted for the increased risk and death rate from coronary heart disease (CHD) deaths from premenopausal RRSO. The risk of CHD is increased in premenopausal women who do not receive hormone replacement therapy after undergoing premenopausal oophorectomy.^27,28^

Our individual-level simulation model follows up 1 000 000 cancer-free individuals aged 30 to 35 years in the US who can potentially move among various health states. Individuals who carry pathogenic variants may develop germline breast cancer (linked to *BRCA1*, *BRCA2*, or *PALB2* genes) or germline ovarian cancer (linked to *BRCA1* or *BRCA2* genes), or they may remain cancer free. Those without these variants may develop sporadic breast or ovarian cancer or remain cancer free. Every individual has a probability of experiencing background all-cause mortality. For identified carriers of a *BRCA1, BRCA2, *or* PALB2* pathogenic variant, undetected pathogenic variant carriers, and noncarriers, disease progression is the same between the population-based testing arm and family history–based testing arm. The probability for disease progression depends on the interventions they receive and their baseline risk.

The model tracked health care decisions, clinical events, and health care costs throughout each woman’s lifetime, with a discount rate of 3% annually for both health outcomes and costs. Our analysis was conducted from a payer’s perspective, which considered only direct medical care costs. We used a lifetime time horizon to capture the long-term benefits of genetic testing and cancer prevention strategies. All costs were adjusted to 2022 US dollars.

Table 1 displays values of modeling parameters and the corresponding data source^22,26,27,29,30,31,32,33,34,35,36,37,38,39,40,41,42,43,44,45,46,47,48,49,50,51,52,53,54,55,56,57,58,59,60,61^ and presents the probability of each pathway. To determine the age-specific incidence of sporadic breast and ovarian cancer, we collected data from US Cancer Statistics 2019 for the general population. Information on the age-specific incidence of breast and ovarian cancer in *BRCA1* or *BRCA2* carriers, breast cancer in *PALB2* carriers, and contralateral breast cancer after an initial diagnosis is gathered from the published literature.^62,63^ Cost parameters included genetic testing, genetic counseling before and after testing, breast and ovarian cancer (by phase of care), and excess CHD. We did not include future health care expenses unrelated to breast cancer, ovarian cancer, or CHD. To provide a lifetime perspective, our study considered the long-term implications and risks. Cancer mortality was derived from the Surveillance, Epidemiology, and End Results Program. We modeled survival after breast and ovarian cancer diagnosis using 10-year survival data. We estimated life expectancy for women without breast or ovarian cancer using female life tables from the National Center for Health Statistics. Our main outcome measure was quality-adjusted life-year (QALY), with a health state utility of 1.0 assigned to healthy individuals undergoing mammography with or without MRI. We obtained utilities for breast and ovarian cancer from the published literature^26,29^ and assigned lower utility for the risk-reduction operations (RRM and RRSO) during the year they were performed. In recognition of the potential psychological impact of receiving a positive genetic test result, we assumed a 1-time disutility of −0.1 in year 1 (utility score, 0.9), whereas previous models reported disutilities ranging from −0.13 to −0.05.

**Table 1.** State-Transition Probabilities, Costs, and Utility Scores in the Microsimulation Model

Parameter	Finding	Source
State-transition probabilities (95% CI)		
Prevalence of *BRCA1* or *BRCA2* pathogenic variant carriers	0.0067 (0.0059-0.0077)	Jervis et al,^29^ 2015
Prevalence of *PALB2* pathogenic variant carriers	0.0021 (0.001-0.0032)	Cybulski et al,^30^ 2015; Manchanda et al,^31^ 2018; Slavin et al,^22^ 2017; Thompson et al,^32^ 2015
Prevalence of positive family history	0.0098 (0.0047-0.0179)	Manchanda et al,^31^ 2018; Manchanda et al,^33^ 2020
Prevalence of *BRCA1* or *BRCA2* pathogenic variant carriers in family history–positive individuals	0.1	
Reduction in breast cancer risk from RRM alone in unaffected carriers	0.91 (0.62-0.98)	Rebbeck et al,^34^ 2004
Reduction in breast cancer risk from RRM and RRSO in unaffected carriers	0.95 (0.78-0.99)	Rebbeck et al,^34^ 2004
Reduction in breast cancer risk from RRSO alone in unaffected carriers	0.51 (0.35-0.63)	Domchek et al,^35^ 2010; Heemskerk-Gerritsen et al,^36^ 2015; Rebbeck et al,^37^ 2009
Reduction in ovarian cancer risk from RRSO in unaffected carriers	0.79 (0.61-0.88)	Rebbeck et al,^37^ 2009
Reduction in breast cancer risk from chemoprevention in unaffected carriers	0.29 (0.17-0.4)	Cuzick et al,^38^ 2015
Annual excess risk of developing CHD after RRSO without HRT	0.0072 (0.0068-0.0076)	Parker et al,^27^ 2013
Cumulative mortality from CHD after RRSO without HRT	0.0303 (0.011-0.043)	Parker et al,^27^ 2013
Uptake of RRM in unaffected carriers	0.47 (0.34-0.56)	Evans et al,^39^ 2009
Uptake of RRSO in unaffected carriers	0.55 (0.45-0.64)	Manchanda et al,^40^ 2012
Uptake of chemoprevention in unaffected carriers	0.163 (0.136-0.19)	Smith et al,^41^ 2016
Compliance of HRT	0.8 (0.76-0.83)	Read et al,^42^ 2010
**Costs, US$** ^a^		
Genetic counseling	50	Schwartz et al,^43^ 2014
Genetic testing	300	Nebula Genomics,^44^ 2023; Color Health,^45^ 2023; Wong et al,^46^ 2023
RRM	24 953	Del Corral et al,^47^ 2015; Guzauskas et al,^48^ 2020; Nelson et al,^49^ 2019; Sun et al,^26^ 2019
RRSO	9566	Sun et al,^26^ 2019; Grann et al,^50^ 2011; Williams-Frame and Carpenter,^51^ 2009
Chemoprevention	5074	Sun et al,^26^ 2019; Grann et al,^50^ 2011
General screening for breast cancer^b^	1801	Sun et al,^26^ 2019; Siu and US Preventive Services Task Force,^52^ 2016
Enhanced screening for breast cancer^c^	39 384	Sun et al,^26^ 2019; Grann et al,^50^ 2011
Breast cancer diagnosis and initial treatment (sporadic, *PALB2*)	101 619	Sun et al,^26^ 2019; Grann et al,^50^ 2011
Breast cancer diagnosis and initial treatment (*BRCA1* or *BRCA2*)	94 388	Sun et al,^26^ 2019; Grann et al,^50^ 2011
Breast cancer follow-up and adjuvant treatment: annual cost in years 1-5 (sporadic, *PALB2* and *BRCA1* or *BRCA2*)	9083	Grann et al,^50^ 2011; Robertson et al,^53^ 2011; Sun et al,^26^ 2019
Ovarian cancer diagnosis and initial treatment	150 240	Sun et al,^26^ 2019; Grann et al,^50^ 2011
Ovarian cancer treatment and follow-up: annual cost in years 1-5	16 517	Sun et al,^26^ 2019; Grann et al,^50^ 2011
Breast cancer terminal care^d^	76 770	Sun et al,^26^ 2019; Grann et al,^50^ 2011
Ovarian cancer terminal care^e^	104 965	Sun et al,^26^ 2019; Grann et al,^50^ 2011
CHD	221 744	Centers for Disease Control and Prevention,^54^ 2018; Heidenreich et al,^55^ 2011; Parker et al,^27^ 2013; Sun et al,^26^ 2019; Tsao et al,^56^ 2022
Fatal CHD^f^	27 012	Sun et al,^26^ 2019; Afana et al,^57^ 2015
**Utility score**		
Test positive for *BRCA1*, *BRCA2*, or *PALB2* (year 1)	0.9	Guzauskas et al,^48^ 2020; Grann et al,^50^ 2011; Eccleston et al,^58^ 2017; Li et al,^59^ 2017
RRM (year 1)	0.88	Sun et al,^26^ 2019; Grann et al,^50^ 2011
RRSO (year 1)	0.95	Sun et al,^26^ 2019; Grann et al,^50^ 2011
Early stage of new diagnosis breast cancer	0.71	Sun et al,^26^ 2019
Advanced stage of new diagnosis breast cancer	0.65	Sun et al,^26^ 2019
Recurrent breast cancer	0.45	Sun et al,^26^ 2019
Remittent breast cancer	0.81	Sun et al,^26^ 2019
End-stage breast cancer	0.16	Sun et al,^26^ 2019
Early-stage of new diagnosis ovarian cancer	0.81	Havrilesky et al,^60^ 2009
Advanced stage of new diagnosis ovarian cancer	0.55	Havrilesky et al,^60^ 2009
Recurrent ovarian cancer	0.5	Havrilesky et al,^60^ 2009
Remittent ovarian cancer	0.83	Havrilesky et al,^60^ 2009
End-stage ovarian cancer	0.16	Havrilesky et al,^60^ 2009
CHD	0.84	van Kempen et al,^61^ 2011

Abbreviations: CHD, coronary heart disease; HRT, hormone replacement therapy; RRM, risk-reducing mastectomy; RRSO, risk-reducing salpingo-oophorectomy.

^a^ Costs are inflated using the medical component of the US Consumer Price Index to 2022 US dollars.

^b^ Cost of mammography every 2 years between 50 and 74 years of age.

^c^ Cost is based on annual MRI with mammography starting at 30 years of age and continuing until 75 year of age.

^d^ Cost includes only costs that are directly associated with care for breast cancer. Health care costs that are not directly associated with breast cancer were not considered.

^e^ Cost includes only costs that are directly associated with care for ovarian cancer. Health care costs that are not directly associated with ovarian cancer were not considered.

^f^ Cost of a fatal myocardial infarction.

### Statistical Analysis

In the microsimulation model, 1 000 000 cancer-free female individuals aged 30 to 35 years enter the model and can move between various health states based on the state-transition probability. We performed internal validation of the model (descriptive, technical, and face validity; eMethods 1 in Supplement 1).^64,65^ Our main outcome measure was the incremental cost-effectiveness ratio (ICER; ie, cost per QALY gained), with the commonly used societal willingness-to-pay threshold of $100 000 per QALY in the US as the cost-effectiveness threshold.^66,67^ The ICER was calculated by dividing the difference in mean lifetime costs by the difference in mean lifetime effects (QALYs) between the 2 strategies: (cost of population-based testing − cost of family history–based testing) / (effect of population-based testing − effect of family history–based testing).

To examine the uncertainty of model parameters, we conducted deterministic 1-way sensitivity analyses and probabilistic analysis. In the 1-way analysis, we altered each parameter one at a time to determine its impact on the results. We varied parameters of probabilities and utility scores within their 95% CIs or ranges where available or by ±10% and costs by ±30%. For probabilistic sensitivity analysis, we varied all model parameters simultaneously across their distributions. We assigned costs a γ distribution, utility score a log-normal distribution, and probability a β distribution.^68^ We then ran 2000 iterations of simulation, generating 2000 estimates of incremental costs and incremental effects by sampling from each parameter’s distribution. Last, we plotted the cost-effectiveness acceptability curve to demonstrate the probability of population-based testing being cost-effective at various levels of willingness-to-pay thresholds.

We also performed the following scenario analyses to explore the effect of extreme values of the following model parameters on the findings: (1) Cost of multigene panel testing varies from $100 to $1000, which allowed us to determine the cost of multigene panel testing at and above which the cost-effectiveness of population-based testing starts to become unfavorable; (2) genetic test uptake rate of 0.3 to 1; (3) older median age at RRM (42 years) and RRSO (46 years); (4) RRM uptake rate of 0; (5) RRSO uptake rate of 0; (6) lower reduction in breast cancer risk from RRM alone (0.5); (7) no reduction in breast cancer risk from RRSO alone; (8) lower reduction in ovarian cancer risk from RRSO (0.5); and (9) 1-year disutility for receiving a positive genetic test result of −0.2 to 0. We also presented approaches to engagement with patients and others affected by the study in eMethods 2 in Supplement 1.

## Results

We simulated 1 000 000 young women aged 30-35 years in the US. In the base case, population-based multigene testing was more cost-effective compared with family history–based testing, with an incremental cost of $469 (95% CI, $443-$495) per woman, an incremental QALY of 0.0085 (95% CI, 0.0072-0.0097) per woman, and an ICER of $55 548 per QALY (95% CI, $47 288-$65 850 per QALY). Compared with the family history–based strategy, population-based multigene testing would prevent an additional 1338 cases of breast cancer and 663 cases of ovarian cancer, but it would also result in 69 cases of excess CHD and 10 excess CHD deaths per million women.

**Figure 2.**
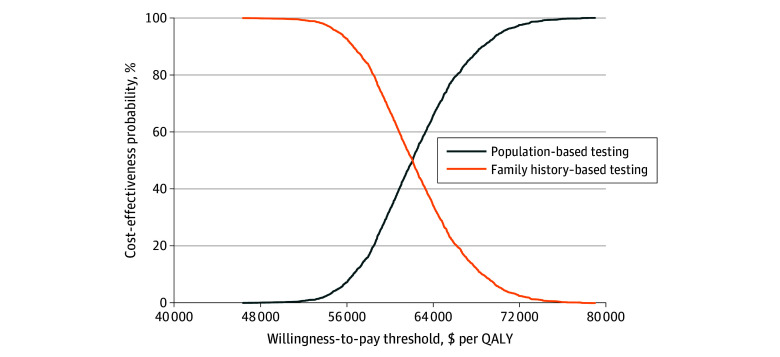
Cost-Effectiveness Acceptability Curve For Sensitivity Analysis We varied all model parameters simultaneously across their distributions for probabilistic sensitivity analysis. We generated 2000 estimates of incremental costs and incremental effects by sampling from each parameter’s distribution to perform probabilistic sensitivity analysis (2000 iterations). A cost-effectiveness acceptability curve was created by plotting the outcomes of 2000 simulations. The curves illustrate the percentage of simulations that showed population-based testing or family history–based testing to be cost-effective at varying willingness-to-pay thresholds. QALY indicates quality-adjusted life-year.

**Figure 3.**
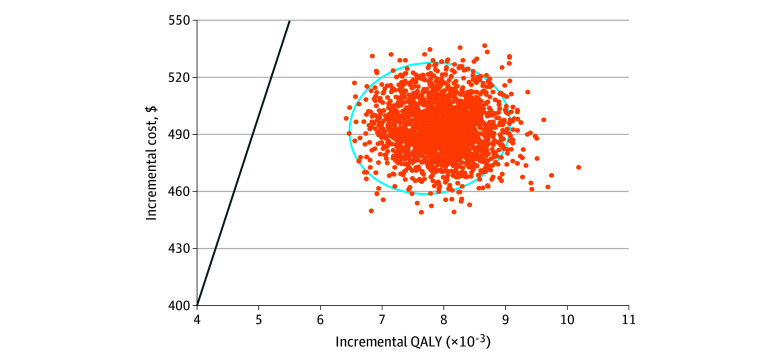
Scatterplot for Incremental Lifetime Costs and Effects of Population-Based Multigene Panel Testing Compared With Family History–Based Testing From Probabilistic Sensitivity Analysis We generated 2000 estimates of incremental costs and incremental effects by sampling from each parameter’s distribution to perform probabilistic sensitivity analysis (2000 simulations). The result of each simulation or iteration in the probabilistic sensitivity analysis (2000 iterations) is represented as a point on the cost-effectiveness plane, forming a “cloud” of potential outcomes. The circle signifies the 95% credible interval of the outcomes. The line indicates the willingness-to-pay threshold of $100 000 per quality-adjusted life-year (QALY).

In 1-way sensitivity analyses, population-based multigene testing remained cost-effective when we varied each parameter individually, indicating that pathogenic variant prevalence, transition probabilities, utility scores, and costs had little individual influence on the result. The probabilistic sensitivity analyses showed that the probability of cost-effectiveness for population-based multigene testing was 100% for all the simulations when using the $100 000 per QALY willingness-to-pay threshold (Figure 2). Figure 3 shows 2000 estimates of incremental costs and incremental effectiveness by sampling from each parameter’s distribution for probabilistic sensitivity analysis.

In scenario analyses, when the cost of the test exceeded $825, population-based multigene testing was no longer cost-effective (ICER, $100 005 per QALY; 95% CI, $87 601-$11 6323). The results from scenario analyses are given in Table 2. Even under extreme conditions for other model parameters, population-based testing remained a cost-effective option.

**Table 2.** Scenario Analyses of the Impact of Extreme Values of Model Parameters

Scenario	Cost (95% CI), $ per QALY
Cost of multigene panel testing	
$100	38 058 (32 284-45 507)
$1000	110 439 (96 183-129 282)
Genetic test uptake rate	
0.3	53 157 (42 278-69 810)
1	55 445 (48 831-63 710)
Older median age at RRM (42 y) and RRSO (46 y)	90 010 (72 248-118 226)
RRM uptake rate of 0	82 468 (66 468-107 119)
RRSO uptake rate of 0	144 827 (105 870-226 925)
Reduction in breast cancer risk from RRM alone (0.5)	61 284 (52 197-73 494)
No reduction in breast cancer risk from RRSO alone	54 198 (46 606-64 117)
Reduction in ovarian cancer risk from RRSO (0.5)	66 916 (56 557-81 205)
One-year disutility for receiving a positive genetic test result	
−0.2	57 610 (49 189-68 820)
0	51 071 (44 206-59 891)

Abbreviations: QALY, quality-adjusted life-year; RRM, risk-reducing mastectomy; RRSO, risk-reducing salpingo-oophorectomy.

## Discussion

This study evaluated the cost-effectiveness of population-based multigene testing, compared with family history–based testing, as a preventive measure for hereditary breast and ovarian cancer using a state-transition microsimulation model. The findings showed that population-based multigene testing was more cost-effective. Compared with the current standard of care, which is family history–based testing, population-based testing led to fewer breast and ovarian cancer cases, but it also resulted in a small increase in CHD cases and deaths.

Variants of unknown significance (VUSs) are associated with unclear risks of cancer and add complexity and uncertainty to the results, potentially causing unnecessary anxiety and leading to inappropriate medical interventions. Current guidelines^23,69^ recommend that VUSs should not be used for guiding medical decisions. A recent systematic review and meta-analysis^70^ found that medical management was similar between individuals with VUSs and benign or likely benign results as more health care professionals become familiar with the uninformative nature of VUSs and VUS-related clinical management guidelines. However, VUSs, being uncertain and not definitively linked to disease risk, can create unnecessary anxiety and add complexity and uncertainty to the results. Investigators have proposed not reporting VUSs in population-based genetic screening settings^71^ because screening aims to identify individuals with known and actionable genetic variations linked to specific conditions who may benefit from further diagnostic testing or interventions. Ethically, it is more responsible to focus on reporting known, clinically relevant findings, balancing the need for precision with the potential harm and confusion that reporting uncertain results may cause.

Findings of this study align with the increasing body of evidence supporting population-based multigene testing. Previous research^14,15,16^ has found that the family history–based testing strategy missed a significant number of pathogenic variant carriers. Population-based multigene testing provides more comprehensive information and improves the early identification of at-risk individuals. Our study differs from the research conducted by Manchanda et al^31^ on the cost-effectiveness of population-based testing for *BRCA1, BRCA2, RAD51C *(OMIM 602774)*, RAD51D *(OMIM 602954)*, BRIP1 *(OMIM 605882)*, *and* PALB2* mutations for preventing breast and ovarian cancer in the following ways. First, we built a state-transition microsimulation model for breast and ovarian cancer. Second, we factored in a 1-time disutility of −0.1 in the first year for a positive test result, acknowledging the potential psychological impact of receiving a positive genetic test result. Third, we chose not to report VUS results due to the added complexity and uncertainty they bring to the results. We believe it is more responsible to focus on reporting known, clinically relevant findings, striking a balance between the need for precision and the potential harm and confusion that reporting uncertain results may cause. Fourth, all tested individuals are offered posttest genetic counseling to ensure the proper use of preventive cancer care. Manchanda et al^33^ found that population-based *BRCA* testing is highly cost-effective for high- and upper-middle–income countries and can potentially prevent thousands more breast cancer cases and hundreds more ovarian cancer cases per million women compared with family history–based testing strategy. Guzauskas et al^72^ also found that population genomic screening is cost-effective in US adults younger than 40 years for the prevention of Lynch syndrome, hereditary breast and ovarian cancer syndrome, and familial hypercholesterolemia. In contrast, Guzauskas et al^48^ found that population genomic screening for hereditary breast and ovarian cancer is moderately cost-effective for younger women. They found that nonadherence to cancer screening by noncarriers and uptake of risk reduction procedures at older age would limit the potential benefits associated with genetic screening and render population-based screening not cost-effective. These findings underscore the importance of posttest counseling for noncarriers to prevent decreased cancer screening and early identification of carriers and early adoption of risk reduction procedures (RRM and RRSO). Both RRM and RRSO do not appear to have a significant adverse impact on long-term general quality of life.^73,74,75^ However, both RRSO and RRM are usually associated with reduced sexual pleasure.^73,76^ This disadvantage may be compensated by a reduction in perceived cancer risk and reduced anxiety.^73,74,76,77,78^

The study’s findings have important clinical implications. The population-based multigene testing offers a more comprehensive and efficient approach to identify individuals at high risk of hereditary breast and ovarian cancer compared with family history–based testing. Implementation of this strategy can lead to early detection and facilitate informed decision-making and personalized risk management for at-risk individuals. These results offer supporting evidence for practice guidelines and policies to recommend more comprehensive genetic testing strategies to identify pathogenic variant carriers. However, it is crucial to address the potential challenges associated with the increased detection of pathogenic variant carriers, such as the need for enhanced screening and risk-reducing interventions.

### Limitations

There are several limitations to this study. The model parameters, including the prevalence of pathogenic variants and the risks of breast and ovarian cancer, are mainly based on data from White women,^29,30,31,32^ which may limit the generalizability of the findings to other populations, as genetic variations and disease risks can vary across different racial and ethnic groups. Additionally, the study used a relatively low cost of $300 for the multigene panel test. However, scenario analysis was conducted using different cost scenarios ranging from $100 to $1000. The analysis revealed that when the cost reached $825, population-based testing became unfavorable compared with family history–based testing. Furthermore, the study assumed a genetic test uptake rate of 70% but also performed scenario analyses to evaluate the impact of varying uptake rates from 30% to 100%. The findings indicated that population-based testing remained cost-effective across different uptake rates. In addition, we did not account for cascade testing of family members of the proband in the revised manuscript. Cascade testing may only moderately increase the identification of pathogenic variant carriers: approximately 70% of the probands may tell their family members and approximately 20% of informed family members may receive genetic testing^79,80^; we simulated a cohort of 1 000 000 women aged 30 to 35 years, and the proportion of the identified probands who have sisters aged 30 to 35 years in the family history–based testing arm is small. Therefore, we did not account for cascade testing of family members of the proband. Furthermore, we did not account for family history when modeling cancer risk for variant carriers. Cancer risk is much higher for variant carriers who have a family history of breast or ovarian cancer.^62^

## Conclusions

In this economic evaluation of population-based multigene testing, we demonstrated the cost-effectiveness of population-based testing for preventing hereditary breast and ovarian cancer. The findings support the need for a shift toward more comprehensive genetic testing strategies to identify pathogenic variant carriers and enable informed decision-making for personalized risk management.

## Notes

Supplement 1. **eMethods 1.** Internal Validation of the Model **eMethods 2.** Approaches to Engagement With Patients and Others Affected by the Study **eFigure.** Microsimulation Model Structure Showing Identification of a Variant, Receipt of Recommended Preventive Interventions, and Clinical Events

Supplement 2.
Data Sharing Statement
