# Supplementary material for: Cost-Effectiveness of Population-Based Multigene Testing for Breast and Ovarian Cancer Prevention
Source: JAMA Netw Open. 2024 Feb 14;7(2):e2356078. doi: 10.1001/jamanetworkopen.2023.56078 (PMC10867683; doi:10.1001/jamanetworkopen.2023.56078)
Supplement: Supplement 1. — eMethods 1. Internal Validation of the Model eMethods 2. Approaches to Engagement With Patients and Others Affected by the Study eFigure. Microsimulation Model Structure Showing Identification of a Variant, Receipt of Recommended Preventive Interventions, and Clinical Events [file jamanetwopen-e2356078-s001.pdf]

Supplementary Online Content

Guo F, Adekanmbi V, Hsu CD, Berenson AB, Kuo Y-F, Shih Y-CT. Cost-effectiveness of population-based multigene testing for breast and ovarian cancer prevention in the US. *JAMA Netw Open*. 2024;7(2):e2356078. doi:10.1001/jamanetworkopen.2023.56078

eMethods 1. Internal Validation of the Model
eMethods 2. Approaches to Engagement With Patients and Others Affected by the Study
eFigure. Microsimulation Model Structure Showing Identification of a Variant, Receipt of Recommended Preventive Interventions, and Clinical Events

This supplementary material has been provided by the authors to give readers additional information about their work

© 2024 Guo F et al. *JAMA Network Open.*

eMethod 1. Internal validation of the model.

Descriptive validity: our model meticulously incorporates all pertinent elements associated with the dynamics of hereditary and sporadic breast and ovarian cancer, ensuring that no crucial aspects are overlooked. It selectively excludes only those factors that have minimal impact on the model's outcomes and conclusions.

Technical validity: In assessing the technical validity of our breast and ovarian cancer model, we focus on the verification or technical soundness of the model. Through systematic debugging, we identify and rectify any errors or discrepancies in the model's coding, calculations, and algorithms. As an illustration, if we assigned a genetic testing uptake rate of 0 for both the population-based testing and family history-based testing groups, we anticipated an equal count of hereditary breast cancer cases (linked to BRCA1/BRCA2/PALB2) between these two groups. Our observation aligned with this expectation, as we recorded an identical occurrence of cases (4439 instances among 1,000,000 women) in both arms. By addressing these technical intricacies, we establish a robust foundation for the model's functionality, bolstering its precision and trustworthiness.

Face validity: Evaluating the face validity of our breast and ovarian cancer model involves considering the extent to which the model's outputs align with the theoretical underpinnings of the disease and its corresponding medical interventions. This criterion is met when the model generates results that are logically and intuitively consistent with the established understanding of the disease dynamics and the expected impact of medical interventions. We investigated the potential prevention of hereditary ovarian cancer (linked to BRCA1/BRCA2) by considering a scenario with 100% genetic testing uptake in the population-based testing arm, combined with full uptake of risk-reducing salpingo-oophorectomy (RRSO) among carriers of pathogenic variants and a complete reduction (100%) in ovarian cancer risk due to RRSO. Our findings indicated that ovarian cancer cases were entirely averted within the population-based testing arm under these conditions.

eMethod 2. Approaches to engagement with patients and others affected by the study.

We consulted with clinicians, including primary care providers, oncologists, genetic counselors, and medical geneticists, as well as engaged with patient support groups to explore the feasibility and significance of unselected genetic testing for breast and ovarian cancer prevention. While direct patient involvement was not incorporated during the study's design and execution, we plan to share our research findings with patient support groups, clinicians, and the research community following publication acceptance. This dissemination aims to foster broader awareness and discussions, ensuring that patient perspectives and stakeholder input are considered in ongoing conversations about genetic testing and its implications for cancer prevention strategies.

eFigure 1. Microsimulation model structure showing identification of a variant, receipt of recommended preventive interventions, and clinical events.

BC, breast cancer; CHD, coronary heart disease; FH, family history; OC, ovarian cancer; RRSO, risk-reducing salpingo-oophorectomy; RRM – risk-reducing mastectomy.

© 2024 Guo F et al. *JAMA Network Open.*

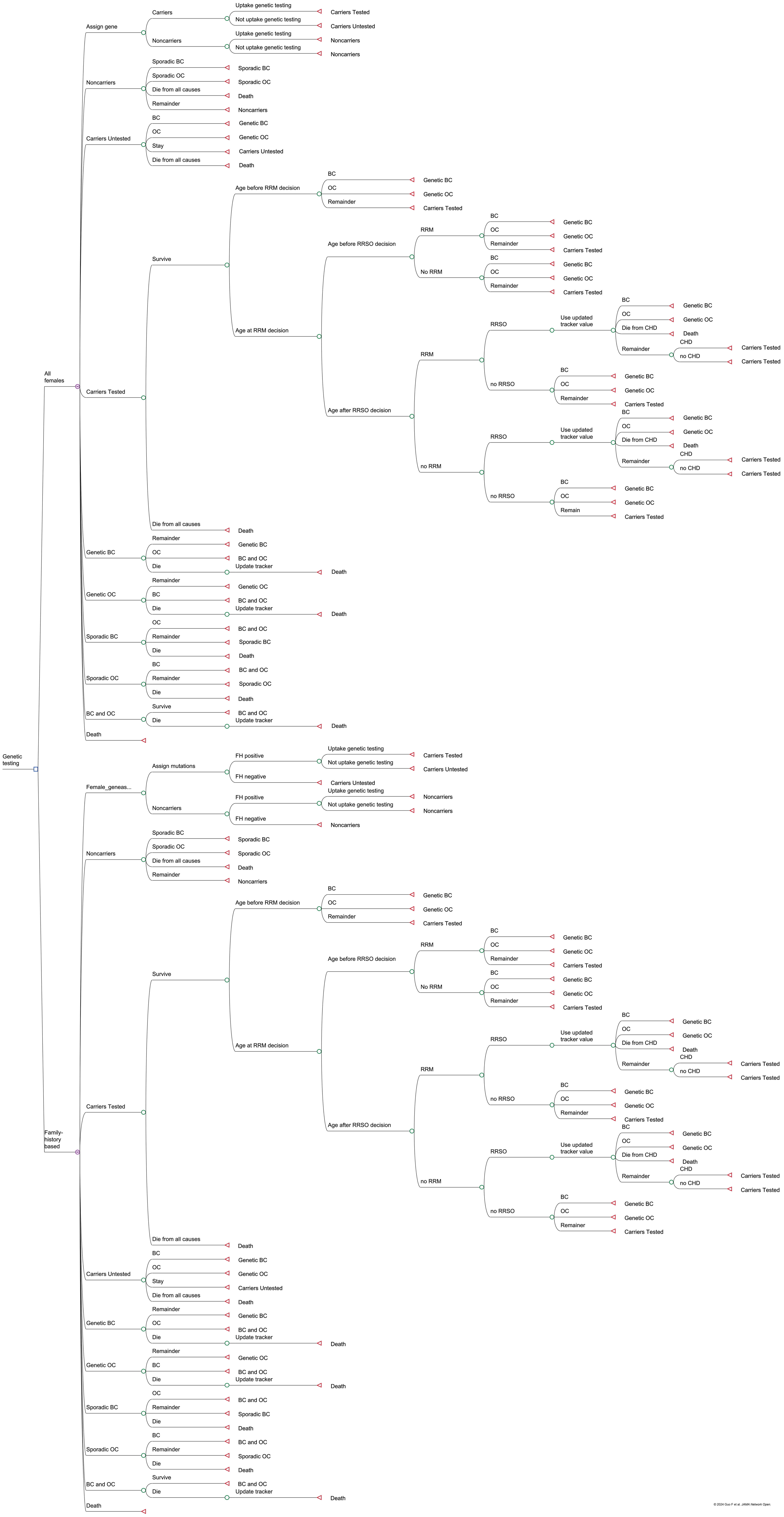

© 2024 Guo F et al. JAMA Network Open.
