# Supplementary material for: Cost-Effectiveness of Population-Based Multigene Testing for Breast and Ovarian Cancer Prevention
Source: JAMA Netw Open. 2024 Feb 14;7(2):e2356078. doi: 10.1001/jamanetworkopen.2023.56078 (PMC10867683; doi:10.1001/jamanetworkopen.2023.56078)
Supplement: Supplement 2. — Data Sharing Statement [file jamanetwopen-e2356078-s002.pdf]

Data Sharing Statement

Guo. Cost-Effectiveness of Population-Based Multigene Testing for Breast and Ovarian Cancer Prevention. *JAMA Netw Open*. Published February 14, 2024.
doi:10.1001/jamanetworkopen.2023.56078

Data
Data available: Yes
Data types: Deidentified participant data
How to access data: Our study used data from several publicly available sources, including United States Cancer Statistics (USCS) database and the Surveillance, Epidemiology, and End Results (SEER) Program. Each of these sources has established procedures for accessing their data. We will direct interested parties to the appropriate contacts to request access.
When available: With publication

Supporting Documents
Document types: None

Additional Information
Who can access the data: Researchers whose proposed use of the data has been approved
Types of analyses: For any purpose
Mechanisms of data availability: with a signed data access agreement
